# Determination of the legal age of majority in Spain and in the European Union. Current situation

**DOI:** 10.4317/jced.62348

**Published:** 2025-01-01

**Authors:** Araceli Miravé, Pedro Abecasis

**Affiliations:** 1 DDS. Head of the postgraduate course in oral and maxillofacial radiology UIC-Barcelona; 2 DDS. Ph.D. Dentist specialist in oral surgery, Professor of radiology at IUEM-Lisbon, Professor of Radiodiagnosis at UIC-Barcelona

## Abstract

**Background:**

Migration brings numerous challenges, especially when undocumented minors arrive in large numbers. Age determination is essential in providing minors with legal protection while preventing abuse of systems designed for their welfare. Traditional methods, such as hand radiographs and orthopantomography, have faced criticism for their lack of precision and reliance on outdated standards. This study evaluates contemporary approaches for determining age, emphasizing the need for updated protocols.

**Material and Methods:**

The study examines the legal framework in Spain regarding age determination of undocumented migrants, emphasizing the phased process involving physical examinations, dental radiographs, and CT scans of the clavicle. The challenges of using traditional methods, including variations in bone maturation across ethnic groups, are addressed.

**Results:**

Analysis revealed limitations in traditional methods such as the Greulich and Pyle atlas and orthopantomography. These methods tend to overestimate age in certain populations and lack applicability in diverse ethnic contexts. CT of the clavicle emerged as a more reliable tool for late adolescents, despite its higher radiation dose.

**Discussion:**

The findings underscore the importance of adapting age estimation methods to diverse populations and leveraging advanced imaging technologies. Radiological techniques must be used judiciously to balance accuracy and ethical considerations. Updating protocols to include multidisciplinary approaches and integrating new technologies can significantly improve outcomes.

**Conclusions:**

Modernizing age determination practices is crucial to align with contemporary needs and protect the rights of migrants. Establishing detailed and specific protocols tailored to diverse populations will ensure both scientific reliability and humanitarian principles in forensic and legal applications.

** Key words:**Forensic Age Estimation,Cone Beam Computed Tomography (CBCT), Mandibular Condyle, Third Molar Apical Closure, Skeletal Maturity, Legal Age Determination, Cortical Bone Analysis, Ethnicity and Age Estimation.

## Introduction

Many of the immigrants arriving at our borders are undocumented and some of them are minors. In 2023, more than 56,000 illegal immigrants arrived in Spain, 5,000 of whom were children. The regulations of the European Union (EU) and of each of the member countries determine the guidelines for action in the face of the massive arrival of immigrants and how to respect their fundamental rights. Minors are taken in and expulsion orders are not enforced against them in most EU countries, or at least not enforced until the migrant has reached the age of majority. The age of majority is set at 18 in all EU countries.

The majority of migrants arriving in Spain come from Central African countries. There are many reasons why many migrants arrive undocumented. Some are tricky in order to pretend to be minors and be accepted by the system. Others because they have never had documentation. In fact, more than 64% of births in sub-Saharan African countries are not registered. The administration of justice has a dual task: on the one hand, to provide legal 

protection, guardianship and care for minors; on the other hand, to prevent abuse of our protection systems.

In Spain, migrant minors enjoy a system of rights and protection under both national legislation and international conventions, with the aim of guaranteeing their welfare, fundamental rights and comprehensive development.

Specifically, minors arriving in Spain enjoy:

-Right to Education: All minors, including undocumented migrants, have the right to access free and compulsory education up to the age of 16. The autonomous regions are responsible for ensuring school places and integration programs to help migrant minors adapt culturally and linguistically.

-Right to Health Care: Migrant minors, regardless of their legal status, have access to public health care. This right guarantees basic and specialised services, and prioritises physical and mental health care for minors in vulnerable situations.

-Special Protection for Unaccompanied Minors (MENAS by its acronym in Spanish): Unaccompanied minors, i.e. those who arrive in Spain without adult guardianship, receive special attention. The public administrations are responsible for their guardianship and for providing accommodation, education and psychosocial care. These minors are placed in specialised centres that promote their integration and well-being.

Spain has developed a robust protection system to guarantee the rights of migrant minors, geared towards their well-being and comprehensive development. However, there are challenges, such as the saturation of minors’ centres in certain regions and the complexity of regularisation processes, which requires a constant effort on the part of the authorities to improve and adapt protection mechanisms.

Age determination is a necessary practice in situations where the age of an undocumented person cannot be confirmed through official documents. This determination is critical because, if a migrant is considered a minor, he or she will have access to legal and social protection that is not granted to an adult. However, the use of radiographic tests and the lack of precision in some procedures, based on studies developed more than 50 years ago, have generated criticism and a call for reform of protocols to protect the fundamental rights of migrants (1-7).

## Material and Methods

Current protocols in age estimation

In Spain, the age determination process is mainly regulated by Organic Law 4/2000 (LOEX) and its associated regulations, which establish that the Public Prosecutor’s Office is responsible for ordering the necessary medical tests to determine the age of an undocumented migrant.

The current protocol in Spain for age determination for legal purposes, in doubtful cases, is carried out, at the request of the Public Prosecutor’s Office, by medical personnel who determine the tests based on their ‘lex artis’ following the criteria set out in the 2011 

document ‘Conclusions of the Working Day on Forensic Age Determination of MENAS. Consensus Document of Good Practice among the Institutes of Forensic Medicine in Spain’ This document lists the different successive and complementary phases until reaching an opinion of majority or minority of age:

Physical examination, interview and morphological examination.

Radiological examination of the carpus of the left hand.

Dental orthopantomography (OPG).

Computed tomography (CT) scan of the proximal epiphysis of the clavicle.

The phases should be carried out consecutively if there are doubts about majority or minority. Once a finding of majority or minority is reached, no further examinations should be carried out.

Medical tests for age determination of undocumented migrants suspected of being minors are carried out by public health professionals. These tests are usually carried out in public hospitals or specialised health centres, under the direction and supervision of forensic doctors or radiologists (5). They should follow the following process:

1. Requesting the tests: Age determination tests are requested by the judicial authorities or the Fiscalía de Menores, in charge of the protection of minors in Spain. When the Police or the Guardia Civil intercept a migrant with doubts about his or her age, they refer the case to the Prosecutor’s Office, which coordinates the procedure and requests the necessary tests (6).

2. Conducting the tests in health centres: Once authorised by the Public Prosecutor’s Office, the tests are carried out in public health centres, usually in hospitals with X-ray equipment and specialists in the interpretation of the results. Radiologists or forensic doctors are responsible for performing the tests, such as wrist X-rays, orthopantomographies (dental X-rays) and, in certain cases, CT scans of the clavicle (7).

3. Evaluation and analysis of results: Forensic physicians compare bone and dental development with reference standards, such as the Greulich and Pyle method for the wrist or orthopantomography for teeth, and issue a technical report indicating the estimated age (8)). These methods are widely used, although they may have a significant margin of error, especially in non-Caucasian populations (9).

4. Sending the results to the Juvenile Prosecutor’s Office: The medical report is sent to the Juvenile Prosecutor’s Office, which reviews the results along with other factors, such as the migrant’s statements or documents submitted. Based on this review, the Prosecutor’s Office determines whether to apply the presumption of minority or, failing that, to establish that the individual is of age (10).

The accuracy of the evidence has generated controversy, as it can have a considerable margin of error and in some cases tends to overestimate the age of minors (11). For this reason, the Supreme Court has ruled that, in situations of doubt, the presumption of minority should be applied, so that the migrant is protected until it is confirmed that he or she is of age (12,13).

Similar medical tests are used in all European countries. However, various criticisms and ethical concerns have led some states such as France and Belgium to seek alternative methods. In Belgium, for example, the National Order of Physicians has expressed reservations about the use of X-rays to determine age, and in France, the verification of official documents before resorting to medical tests is emphasised (11). The process has been criticised for being invasive and not always reliable. Recent legal reforms have sought to introduce a multidisciplinary and holistic approach, as reflected in the Draft Bill regulating these procedures (2).

The medical methods are detailed below:

-Radiological examination of the hand. Greulich and Pyle method

**Figure 1:**
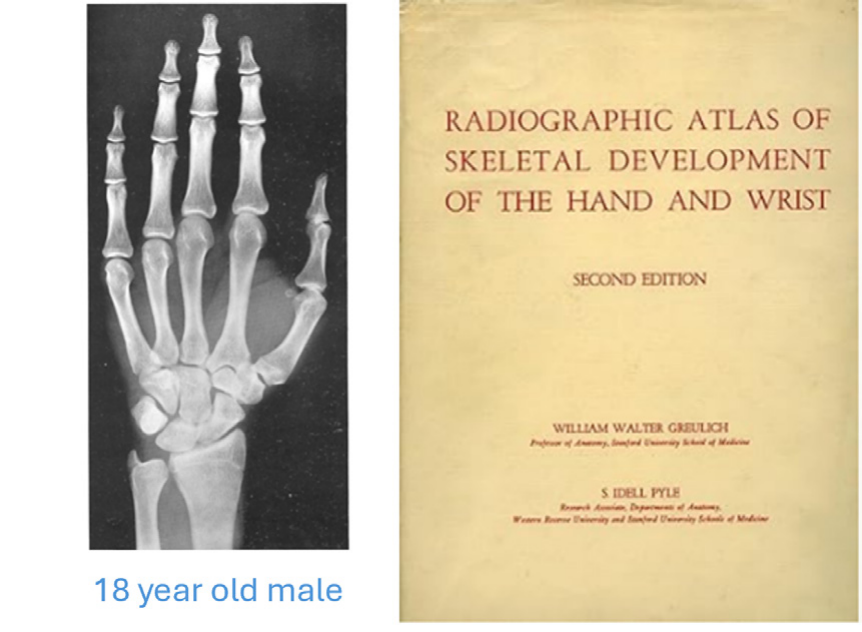
Image of the cover of the second edition of Greulich and Pyle’s Atlas, jointly published by Stanford and Oxford Universities. Left Hand X-ray of an 18-year-old male as a reference image for age assessment (1959).

The Greulich and Pyle method (Radiographic atlas of skeletal development of the hand and wrist, 1959) is one of the most widely used standard procedures for estimating bone age in children. First published in 1959, the method is based on an atlas containing radiographic images of the hand and wrist of white American children between the ages of birth and 18 years. Each image in the atlas (Fig. 1) represents an average of bone maturation for a specific age and allows the observer to compare the individual’s radiograph with reference images to estimate bone age (8).

Limitations of the Method

Greulich and Pyle developed the atlas from a large but geographically limited sample. The technique is considered reliable for determining bone age up to adolescence, as hand bone development follows a well-defined pattern during these growth stages.

The method allows for a quick and practical estimation of bone age, which is especially useful in clinical and forensic contexts. Its ease of application makes it one of the preferred methods in paediatrics and orthopaedics (9).

As a widely validated method used in numerous populations, it has been shown to have good reliability in many regions of the world, although it has been identified that it may have variations in accuracy depending on ethnicity or geographic group (8). The Greulich and Pyle method has been validated in several populations, although its accuracy may vary by ethnicity due to differences in bone growth patterns.

The following are some of the ethnicities and populations in which the method has been used and validated:

-Caucasian population (US and European): The Greulich and Pyle method was originally developed and validated in a sample of Caucasian children in the United States (8), making it particularly reliable in this population.

-Latin American population: Studies in Latin America have shown that the method can be applied effectively, although with certain limitations in accuracy depending on the specific ethnic group. Adjustments have been made in countries such as Brazil and Mexico (14).

-Asian population: It has been tested in children from several Asian countries, such as Japan, Korea and China. In Japan, validation has been favorable, while in Korea and China the method tends to overestimate age compared to local bone maturation (15).

-Middle Eastern and North African population: Application in Middle Eastern populations, including Saudi Arabia, has shown some accuracy, although some studies have recommended adjustments to improve accuracy compared to the reference population (16).

-African population: In Africa, it has been used in countries such as Nigeria and South Africa, although it has been reported that bone maturation in some African ethnicities may differ significantly from the original reference population, affecting the accuracy of the method in these regions (17).

-Indian population: In India, studies have shown that the method can be applied, although there are differences in bone maturation in some regions of the country, leading to specific adjustments to increase accuracy (18).

Each of these studies suggests that while the Greulich and Pyle method is a useful starting point for estimating bone age, accuracy may vary by ethnicity and adjustments may be advisable in some populations to obtain more accurate results.

Advantages of the method

Relevance to pediatric development: Bone maturation is a reliable marker of biological development of individuals, more accurate than chronological age in reflecting growth and development. This is especially useful in children with growth faltering, in whom bone age may indicate the need for medical intervention (19).

Forensic and legal applications: In legal and forensic contexts, the method allows the approximate age of individuals to be established in identification cases and in age studies in migration situations, where age documentation may be insufficient or non-existent (20).

Robust statistical basis: The methodology is based on statistical analysis of a large amount of radiographic data, which supports its validity and makes it reliable for estimating bone age in many infant and juvenile populations (20).

The accuracy of the method is compromised when applied to populations of different ethnicities or with variations in physical developmental conditions, such as differences in nutrition and general health. In addition, the original atlas is based on data from several decades ago, which raises the need for updates to reflect contemporary trends in bone development (20).

Radiation Dose

The wrist X-ray used in this method has a relatively low radiation dose, approximately 0.005 milliSieverts (mSv), 0.001 mSv with digital radiology, which is comparable to the exposure received during a few hours of aeroplane flight. This makes it safe for occasional use in medical and forensic contexts (21).

-Dental Orthopantomography

Orthopantomography, or panoramic dental radiography, is an imaging technique that allows the visualization of the teeth, maxilla and mandible in a single radiograph, assessing the development of the crown and roots of permanent teeth, particularly the third molars. Since the formation of third molars varies between the ages of 17 and 25 years, this technique is used to determine whether an individual has reached the age of majority, because dental development is a reliable indicator of maturation and tends to be less influenced by environmental factors than bone development (22,23).

**Figure 2:**
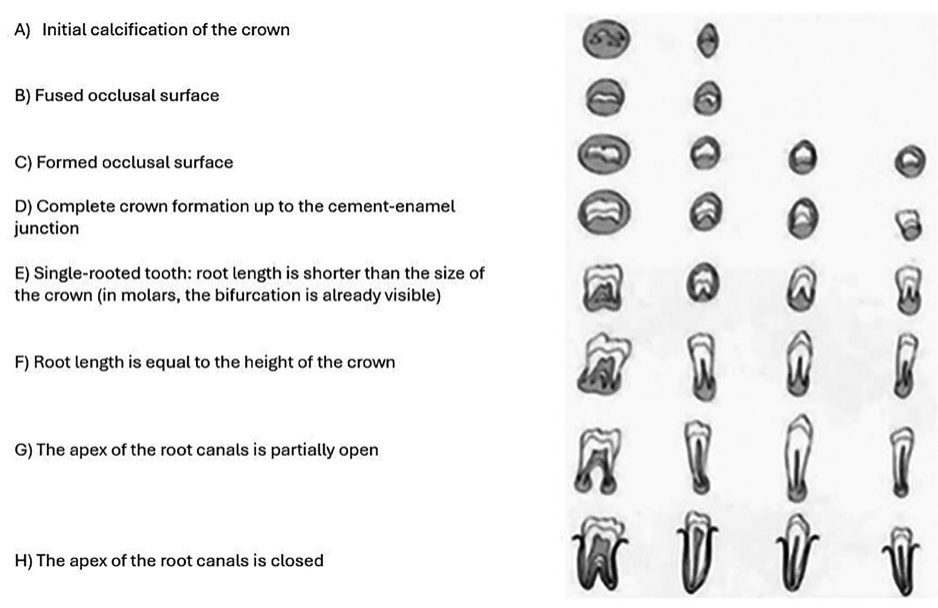
Classification of dental development in eight stages. Demirjian (1973).

Several age determination Tables based on orthopantomography are used. The most representative are:

-Demirjian Table: this method was developed by Demirjian *et al*. in 1973 from a sample of French children and adolescents and is one of the most widely accepted and widely used Tables in age estimation from dental radiographs. Demirjian’s Table classifies the stages of tooth development into eight stages, from ‘A’ to ‘H’, based on the formation and maturation of each mandibular tooth on one side (Fig. 2). These stages are scored and summed to calculate an estimated dental age (24). Although designed for a French population, the Table has been applied in various populations worldwide, albeit with some limitations in accuracy, especially in non-Caucasian populations. For this reason, variants of the Demirjian method exist to better suit different ethnic groups.

**Figure 3:**
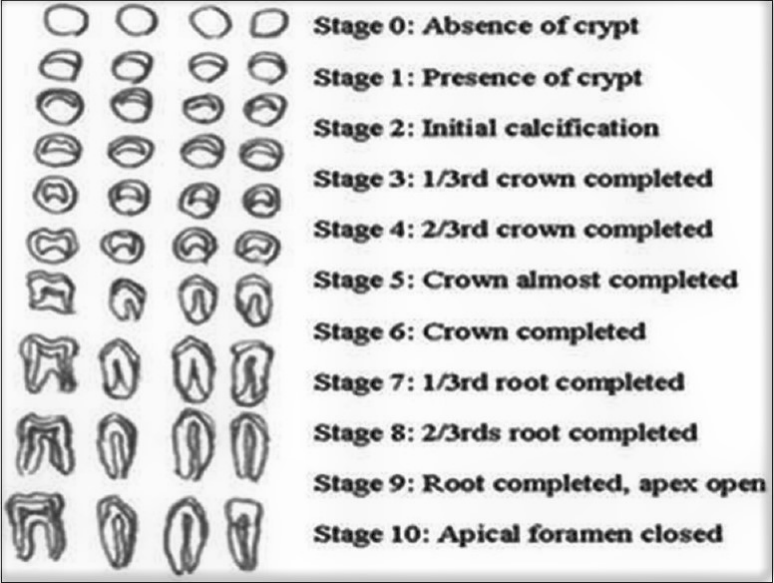
Classification of dental development in ten stages, from the absence of crypt to complete closure of the root apex. (Nolla 1960).

-Nolla Table: Created by Carmen Nolla in 1960, this Table describes 10 stages of tooth development, from the onset of calcification to complete eruption of the tooth (Fig. 3). Each stage is classified numerically, allowing practitioners to derive an estimated age from the stage of development observed in the teeth on the radiograph (25). This method has been used to improve accuracy in populations where the Demirjian method may not be as effective. The Nolla Table has been particularly useful in forensic and archaeological studies to obtain an age estimate in children of different ethnic groups and geographical backgrounds.

**Figure 4:**
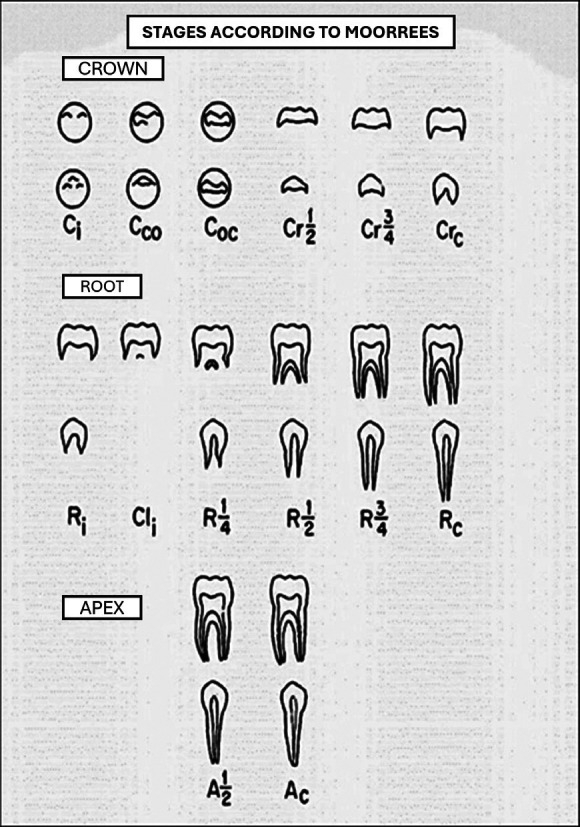
Moorrees, Fanning and Hunt table (1963).

-Moorrees, Fanning and Hunt Table: This method, developed in 1963 from a sample of the US population, proposes a classification of tooth development into several specific stages for each tooth, which allows for a detailed analysis of the maturation of permanent teeth (Fig. 4) (26). Unlike other methods, Moorrees and colleagues classified teeth into 14 stages for crown and root formation, which provides a more accurate and detailed assessment. Although the method has been less popular than Demirjian’s, its use in populations similar to the original (such as the United States and Canada) has proven to be effective and reliable in studies requiring a high level of detail in age determination.

-Willems’ Table: Willems and colleagues adapted Demirjian’s method in 2001 to improve the accuracy of age estimation in populations where it was observed that the original method tended to overestimate age (27). In this approach, Willems adjusted the scores for the developmental stages of the mandibular teeth, resulting in a more accurate version that is applicable in European countries and some Asian populations. This revised method is widely used in age studies in forensic and migration contexts, as it offers greater accuracy in certain groups.

Each of these Tables allows an age estimate to be obtained from the dental development observed in an orthopantomography. However, it is important to bear in mind that dental development may vary according to ethnic group and geographical setting, which means that the choice of chart must be adapted to the specific population under study. In forensic, legal and clinical contexts, it is common to combine more than one chart or make specific adjustments based on ethnicity to improve the accuracy of the estimation. In addition, due to the diversity in dental growth patterns, some studies have recommended additional validations of these methods for use in African, Latin American, and Southeast Asian populations.

In summary, orthopantomography and its study using the Demirjian, Nolla, Moorrees and Willems charts are fundamental tools for age determination in children and adolescents. It is necessary to consider that the development of third molars can vary significantly between individuals and populations. Some individuals may not even develop these teeth at all, which limits the accuracy of estimation in certain cases (22,23). The geographical and ethnic context should be carefully considered, and in some cases, it is advisable to use complementary or adjusted methods to obtain an accurate assessment of biological age.

Radiation Dose

Radiation exposure during orthopantomography is approximately 0.025 to 0.030 mSv, equivalent to three days of background radiation (21).

-Assessment of the clavicle by computed tomography (CT) scanning

**Figure 5:**
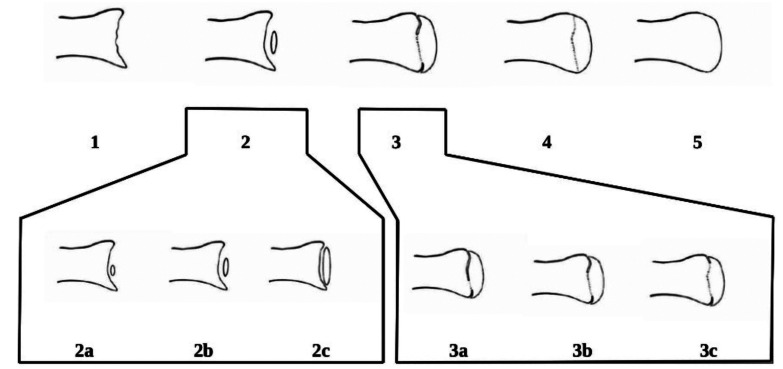
Diagram of maturation stages according to the Schmeling and Kellinghaus classification systems (adapted from Wittschieber *et al*. 2004).

Computed tomography (CT) of the clavicle is an increasingly used method for age estimation, especially in adolescents older than 16 years. The clavicle is one of the last bones to complete its ossification process, which usually ends between the ages of 21 and 25. This method analyses the fusion of the cartilage at the sternal end of the clavicle, which fuses completely later than other bones, allowing an accurate estimation of the age of majority (Fig. 5) (28,30).

CT provides detailed three-dimensional images, allowing assessment of the different stages of ossification of growth plate. It is useful to differentiate between adolescents completing maturation and young adults. Several studies, such as those by Schmeling *et al*. (2004) and Schulz *et al*. (2005) have shown that CT of the clavicle is more accurate than conventional radiographs when assessing the age of majority in late adolescents, as the final fusion of the clavicle may extend up to the age of 25 years (28,30).

Radiation dose

CT of the clavicle involves a higher radiation dose compared to the two previous methods. Exposure is estimated to be around 1.5 to 8.8 mSv, equivalent to 6 months to 3 years of background radiation, significantly higher than a wrist X-ray and orthopantomography combined (21). For this reason, it is recommended that CT is only used when other methods do not provide conclusive results and its use is justified due to clinical or forensic need.

-Total radiation dose of the entire protocol.

**Figure 6:**
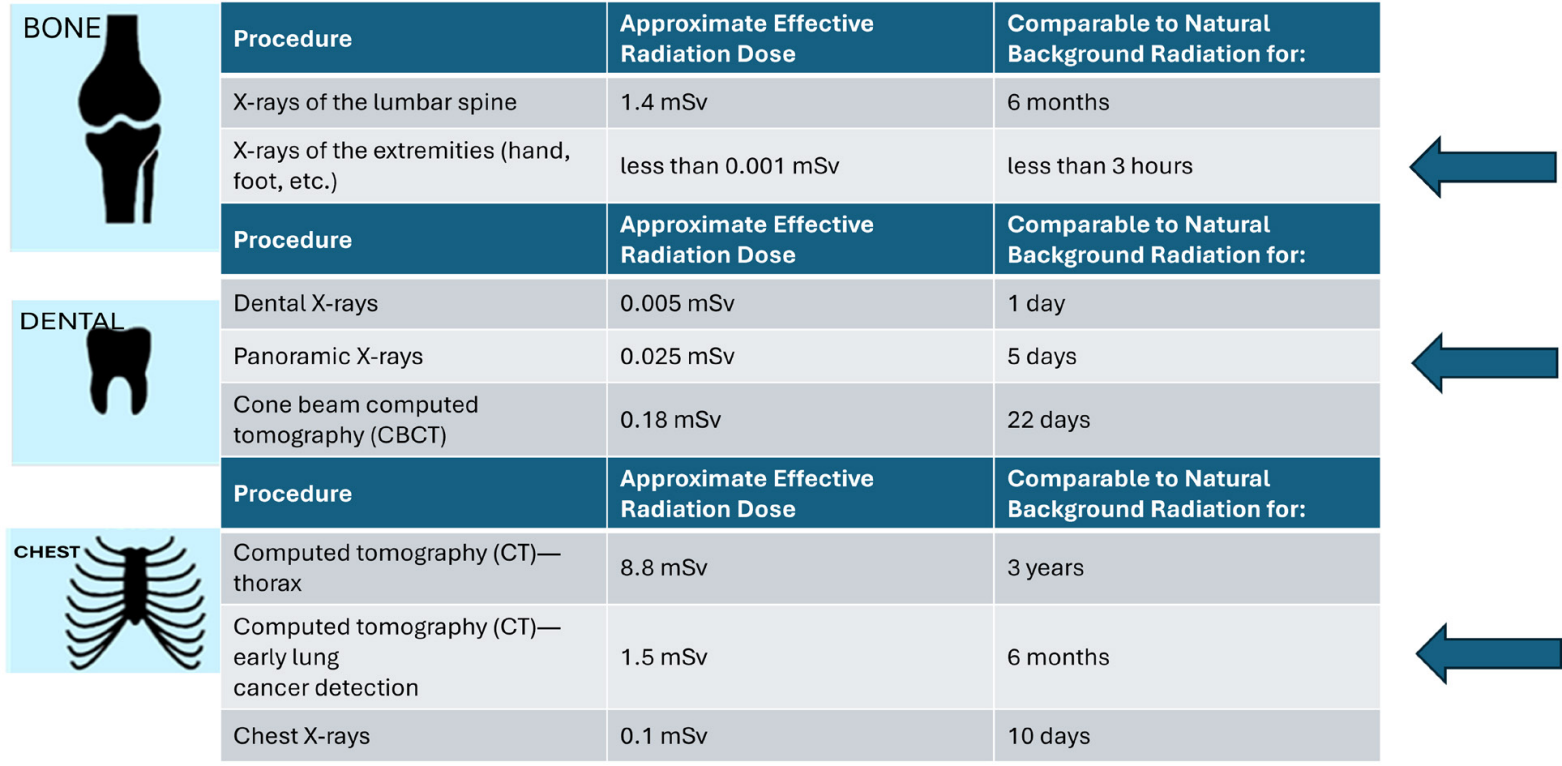
Radiation dose in mSv compared to background radiation days. www.radiolgyinfo.org

The estimated radiation dose from the three standardized tests adds up to approximately between six months and three years of natural background radiation (21), (Fig. 6).

All these tests lead to a diagnosis of majority or minority age, with an unquantified, but estimated very high margin of error (32).

## Conclusions

The debate on age of majority determination for undocumented migrants is open and active, reflecting the need to balance scientific accuracy and respect for human rights. Although current methods in Spain and the EU remain predominantly medical, there is a movement towards multidisciplinary approaches that are more respectful of the dignity of the individual.

We have detected that there is an abundance of legislation that regulates general aspects of action in the case of suspected minors. However, we have not found protocols regulating the details of the procedures. We consider it essential to draw up more detailed and specific protocols for health personnel, not only based on the lex-artis, but also regulating aspects such as.

1. The type of radiological equipment to be used. Not all hospitals in Spain have radiology units equipped with modern technology to minimise radiation doses for the presumed minor.

2. The methods/Tables to be used depending on ethnicity. Not all methods are validated for all ethnicities. In many ethnicities a tendency to overestimate age has been demonstrated.

The protocols are based on studies from more than 50 years ago. They do not consider the evolution of imaging technology, or the many studies carried out in recent years that open the door to assessing other anatomical parameters for age determination, which could represent a significant advance in the accuracy of the reports and in the protection of the individual.

These conclusions highlight the need to update and standardize the methods and resources used in age estimation in the forensic medical field in Spain.

To implement these changes, considerable legislative effort and adequate resources are required (2,5,31).

## Data Availability

The datasets used and/or analyzed during the current study are available from the corresponding author.

## References

[1] Folguera Crespo J, Puerta Ruiz de Azúa C, Moya García S (2022). El procedimiento de determinación de la edad de los menores extranjeros no acompañados. Actualidad Jurídica Uría Menéndez.

[2] (2023). APL procedimiento de evaluación de la edad. Ministerio de Justicia.

[3] Camacho Basallo P (2018). Correlación de la madurez esquelética en base a diversos métodos de determinación en población española. Universidad de Sevilla.

[4] Santonja Medina F, García de la Rubia S, Pastor Clemente A (1998). Valoración de la edad ósea. Su importancia en Medicina del Deporte. Selección.

[5] (2019). Protocolo de actuación para la determinación de la edad en menores extranjeros no acompañados. Consejo General del Poder Judicial.

[6] Fiscalía General del Estado (2020). Circular 1/2020 sobre criterios de actuación para la determinación de la edad de menores extranjeros. Ministerio de Justicia.

[7] Ley Orgánica 4/2000, sobre derechos y libertades de los extranjeros en España y su integración social.

[8] Greulich WW, Pyle SI (1959). Radiographic atlas of skeletal development of the hand and wrist. 2nd ed.

[9] Ontell FK, Ivanovic M, Ablin DS, Barlow TW (1996). Bone age in children of diverse ethnicity. *Am J Roentgenol*.

[10] (2021). Informe sobre la aplicación de la normativa en menores no acompañados. Ministerio de Justicia.

[11] (2018). Age assessment practices in Europe. European Union. European Asylum Support Office.

[12] (2018). Age assessment practices in Europe. European Union. European Asylum Support Office.

[13] Garamendi PM (2011 Jan 1 [cited 2024 May 28]). Conclusiones de la Jornada de Trabajo sobre Determinación Forense de la Edad de los Menores Extranjeros no acompañados. Documento de Consenso de Buenas Prácticas entre los Institutos de Medicina Legal de España. Revista Española de Medicina Legal [Internet].

[14] Bull RK, Edwards PD, Kemp PM, Fry S, Hughes IA (1999). Bone age assessment: a large scale comparison of the Greulich and Pyle, Tanner and Whitehouse (TW2) and Fels methods. *Arch Dis Child*.

[15] Zhang A, Sayre JW, Vachon L, Liu BJ, Huang HK (2009). Racial differences in growth patterns of children assessed on the basis of bone age. *Radiology*.

[16] Alshamrani K, Almazrooa A, Alqahtani Z, Al-Saleh Y (2020). Validation of the Greulich and Pyle Atlas for determination of skeletal age for Saudi children. *Ann Saudi Med*.

[17] South African Medical Research Council (2017). Bone age assessment in South African children. *SAMJ*.

[18] Narayana Gowda BS, Shashikala AK, Suma MR (2013). Applicability of Greulich and Pyle skeletal age standards to Indian children. *J Forensic Leg Med*.

[19] Mughal AM, Hassan N, Ahmed A (2014). Bone age assessment methods: a critical review. *Pak J Med Sci*.

[20] Schmeling A, Olze A, Reisinger W, Geserick G (2004). Age estimation in the forensic context. *Int J Legal Med*.

[21] (2022). Dosis de radiación ¿Qué son los rayos X y qué hacen?. RadiologyInfo.org.

[22] Cameriere R, De Luca S, Biagi R, Cingolani M, Farronato G, Ferrante L (2014). Accuracy of Cameriere's cut-off value for third molar in assessing 18 years of age. *Forensic Sci Int*.

[23] García-Hernández F, Norrie C, Lemos RM (2021). Ethical and legal issues in the age estimation of unaccompanied minors in Europe: A systematic review. J Forensic Legal Med.

[24] Demirjian A, Goldstein H, Tanner JM (1973). A new system of dental age assessment. *Hum Biol*.

[25] Nolla CM (1960). The development of the permanent teeth. *J Dent Child*.

[26] Moorrees CF, Fanning EA, Hunt EE (1963). Age variation of formation stages for ten permanent teeth. *J Dent Res*.

[27] Willems G, Van Olmen A, Spiessens B, Carels C (2001). Dental age estimation in Belgian children: Demirjian's technique revisited. *J Forensic Sci*.

[28] Wittschieber D, Schulz R, Vieth V, Schmidt S, Pfeiffer H, Schmeling A (2010). Enhanced possibilities to make statements on the ossification status of the medial clavicular epiphysis using an amplified staging scheme in evaluating thin-slice CT scans. International Journal of Legal Medicine*.

[29] Rodes F (2022). , Garamendi PM. Determinación de la edad mediante TC de clavículas. A propósito de un caso. Int J Forensic Anthropology and Odontology.

[30] Garamendi PM, Botella MC (2007). Fusión de la epífisis esternal de la clavícula en relación con la edad. Aplicaciones en la estimación forense de la edad. Cuad Med Forense.

[31] (2018). Determinación de la edad de menores extranjeros indocumentados. Defensor del Pueblo.

[32] Pérez Candela V, Pérez Bello C, González Esmoris I (2019). Estimación de la edad cronológica mediante técnicas de imagen. Canarias Pediátrica.

